# Clinical and experimental effects of *Nigella sativa* and its constituents on respiratory and allergic disorders

**Published:** 2019

**Authors:** Zahra Gholamnezhad, Farzaneh Shakeri, Saeideh Saadat, Vahideh Ghorani, Mohammad Hossein Boskabady

**Affiliations:** 1 *Neurogenic Inflammation Research Center, Mashhad University of Medical Sciences, Mashhad, Iran.*; 2 *Department of Physiology, Faculty of Medicine, Mashhad University of Medical Sciences, Mashhad, Iran.*; 3 *Natural Products and Medicinal Plants Research Center, North Khorasan University of Medical Sciences, Bojnurd, Iran.*; 4 *Pharmaceutical Research Center, Mashhad University of Medical Sciences, Mashhad, Iran.*

**Keywords:** Nigella sativa, Thymoquinone, Experimental effect, Clinical effect, Respiratory diseases, Allergic diseases

## Abstract

**Objective:**

Black cumin or *Nigella sativa* (*N. sativa*) seed has been widely used traditionally as a medicinal natural product because of its therapeutic effects. In this review, the medicinal properties of *N. sativa* as a healing remedy for the treatment of respiratory and allergic diseases, were evaluated.

**Material and Methods:**

Keywords including *Nigella sativa*, black seed, thymoquinone, respiratory, pulmonary, lung and allergic diseases were searched in medical and nonmedical databases (i.e. PubMed, Science Direct, Scopus, and Google Scholar). Preclinical studies and clinical trials published between 1993 and 2018 were selected.

**Results:**

In experimental and clinical studies, antioxidant, immunomodulatory, anti-inflammatory, antihistaminic, antiallergic, antitussive and bronchodilatory properties of *N. sativa* different extracts, extracts fractions and constituents were demonstrated. Clinical studies also showed bronchodilatory and preventive properties of the plant in asthmatic patients. The extract of *N. sativa* showed a preventive effect on lung disorders caused by sulfur mustard exposure. The therapeutic effects of the plant and its constituents on various allergic disorders were also demonstrated.

**Conclusion:**

Therefore, *N. sativa* and its constituents may be considered effective remedies for treatment of allergic and obstructive lung diseases as well as other respiratory diseases.

## Introduction

During recent decades, environmental factors (e.g. diet and pollutants) as well as individual factors (e.g. genetics and epigenetics) resulted in an increase in the incidence of inflammatory, allergic and immunodeficiency diseases. An imbalanced immune system would induce several pathological mechanisms such as the release of cytokines and inflammatory mediators, recruitment of inflammatory cells, apoptosis, dysfunction of cell repair processes and oxidative stress. These mechanisms may lead to a cascade of inflammatory and destructive processes producing the pathological manifestations of allergic diseases such as asthma (Balkissoon et al., 2011 ▶). 

Prevention, treatment and management of allergic and immunodeficiency diseases including bronchial asthma, hay fever, sinusitis, arthritis, and inflammatory bowel disease need new approaches affect such pathological mechanisms and balance the immune system (Gholamnezhad et al., 2015a ▶). Black cumin or *Nigella sativa* (*N. sativa*) seed has been widely used traditionally as a food ingredient and herbal remedy for treatment of many inflammatory and allergic diseases in African, Arab and Indian nations especially in the south west of Asia (Tembhurne et al., 2011 ▶). By now, many active ingredients have been identified and isolated from* N. sativa* of different varieties. Oil, carbohydrates, protein, fiber, ash, and saponins are generally present in *N. sativa* seed. There are trace amounts of non-oily and non-caloric components with pharmacological effects in the plant seed. These constituents are phyto-alkaloids, including pyrazol (nigellicine and nigellidine), isoquinoline (nigellicimine and nigellicimine-N-oxide) as well as flavonoid (comferol), diglucoside and digalactoside, alpha-hederin, saponins, vitamins (riboflavin, pyridoxine, niacin, thiamin, folic acid and vitamin E) and minerals (sodium, potassium, calcium, magnesium, copper, iron , and phosphorus) (Hussein El-Tahir and Bakeet, 2006 ▶; Nickavar et al., 2003 ▶).

The *N. sativa *oil is comprised of linoleic, palmitic, oleic, dihomolinoleic, and eicosadienoic acids (Al-Jassir, 1992 ▶; Ali and Blunden, 2003 ▶; Nickavar et al., 2003 ▶). There are several pharmacologically active constituents in the essential oil of the plant, including thymoquinone (TQ), thymohydroquinone, dithymoquinone, p-cymene, carvacrol, 4-terpineol, t-anethol, sesquiterpene longifolene, α -pinene and thymol (Ahmad et al., 2013 ▶). According to experimental and clinical studies, these constituents make *N. sativa* seed a valuable herbal remedy for treatment of various disorders (Gholamnezhad et al., 2016 ▶; Norouzi et al., 2018 ▶; Shakeri et al., 2016 ▶). The therapeutic effects of the plant extract against hypertension, diabetes and metabolic syndrome complications (e.g. obesity, dyslipidemia, and high blood glucose), cyclic mastalgia (analgesic effects), hand eczema, vitiligo, pediatric seizures, opioid dependence, anxiety, infectious diseases (e.g. infections caused by human immunodeficiency virus, hepatitis C virus, and *Helicobacter pylori*), infertility, asthma, chemical war injuries, tonsillopharyngitis, allergic rhinitis, rheumatoid arthritis, dyspepsia, celiac disease, and hepatotoxicity of methotrexate were demonstrated in clinical studies (Ahmad et al., 2013 ▶; Gholamnezhad et al., 2016 ▶; Tavakkoli et al., 2017 ▶). 

In traditional medicine, *N. sativa *alone or in combination with honey was used to ameliorate respiratory diseases such as chest congestion, bronchospasm and asthma (Ave-Sina, 1990 ▶). In pharmacological studies, the plant constituents like TQ, dithymoquinone, nigellidine, nigellicine, carvacrol, and thymol were introduced as effective ingredients of the plant (Ahmad et al., 2013 ▶).

Anti-asthmatic activity of nigellone in bronchitis and asthma was shown (Wienkotter et al., 2008) and it was proposed that such effect may be due to nigellone inhibitory effect on the release of histamine from the mast cells (Chakravarty, 1993 ▶) and 5-lipoxygenase pathway in granulocytes (El-Dakhakhny et al., 2002). According to the literature, TQ is the main component of *N. sativa*, and is responsible for most of plant’s biological activities (Woo et al., 2012 ▶). The effects of TQ on antioxidant enzymes and reactive oxygen species systems, pro-inflammatory mediators/cytokines, inflammatory signaling pathways including nuclear factor kappa B (NF-κB), signal transducer and activator of transcription 3 (STAT3), mitogen-activated protein kinase (MAPK), peroxisome proliferator-activated receptor gamma (PPAR-γ), and protein kinase B (AKt), and apoptosis indicated *N. sativa* effectiveness in prevention and treatment of allergic and inflammatory diseases (Woo et al., 2012 ▶). The aim of this review is to report, and compare clinical evidence on therapeutic effects of *N. sativa* in allergic and respiratory diseases. 

## Materials and Methods

Online databases including PubMed, Science Direct, Scopus, and Google Scholar were searched for studies published between 1993 and the end of October 2018 using the following combination of terms in the title and abstract: (*Nigella sativa* OR *N. sativa* OR black seed OR thymoquinone) AND (Respiratory OR Pulmonary OR lung OR allergic diseases). 

## Results

**The effect of** ***N. sativa*** **and its constituents on allergic disorders**

The anti-allergic effects of* N. sativa* and its constituents were evaluated in experimental and clinical studies.

**Experimental evidence**

The anti-inﬂammatory effects of TQ in a rat model of allergic rhinitis were examined; it was indicated that TQ treatment (3 and 10 mg/kg, intraperitoneally (i.p.), for 21 days) decreased interleukin (IL)-4 and immunoglobulin E (IgE) levels, and suppressed the expression of tumor necrosis factor-α (TNF-α) and IL-1β. It also reduced eosinophil infiltration and edema in the nasal mucosa (Günel et al., 2017 ▶).

To evaluate the anti-allergic effect of TQ, the systemic anaphylactic shock technique using the compound 48/80 (A synthetic compound that induces histamine release from mast cells), was performed. Results showed that treatment with TQ (50 and 100 mg/kg, i.p., for 5 days) significantly reduced TNF-α and IL-1β in both bronchoalveolar lavage fluid (BALF) and lung tissue homogenates and decreased histamine release in rat peritoneal mast cell preparation (RPMCs), (El Aziz et al., 2011a ▶; El Aziz et al., 2011b ▶).

The anti-inﬂammatory effect of TQ (3mg/kg, i.p., for 5 days) in allergic lung inflammation was demonstrated. It was reported that TQ decreased IL-4, IL-5, and IL-13 but increased IFN-γ in BALF and lung homogenates. It also reduced serum levels of total IgE as well as ovalbumin (OVA)-specific IgE, IgG1 and IgG2a and diminished the numbers of eosinophil infiltrates and goblet cell hyperplasia in the lung tissue (El Gazzar et al., 2006a ▶). Administration of TQ (3 mg/kg, i.p., for 5 days) to mice with allergic airway inflammation induced by OVA, showed that TQ decreased IL-4, IL-5, and IL-13 in BALF and reduced lung cell and prostaglandin D_2_ (PGD_2_) production, cyclooxygenase-2 (COX-2) expression, peribronchial eosinophil infiltration and the goblet cells hyperplasia in the airways (El Mezayen et al., 2006 ▶). Similarly, administration of TQ (3 mg/kg, i.p.) for the 5 days preceding the first challenge with OVA in sensitized mice, inhibited 5-lipoxygenase and reduced total and differential cell counts, the levels of leukotrienes (LT) B4 and C4, IL-4, IL-5, IL-13, and IL-10 in BALF (El Gazzar et al., 2006b ▶). The experimental evidence on anti-allergic effects of *N. sativa* and its constituents are summarized in Table 1a.

**Table 1a:** The experimental evidence on anti-allergic effects of *Nigella sativa* and its constituents

**Plant preparations**	**Dose/route of administration/** **treatment period**	**Study models**	**Effects**	**Reference**
TQ	3 and 10mg/kg, i.p., for 21 days	OVA-induced allergic rhinitis in rat	Decreased IL-4, IgE, TNF-α, and IL-1β levels and reduced eosinophil infiltration and edema	(Günel et al., 2017 ▶)
TQ	50 and 100mg/kg, i.p., for 5 days	Allergy induced by the synthetic compound 48/80 in rats	Reduced TNF-α and IL-1β in both BALF and lung tissue homogenates and decreased histamine release from RPMCs	(El Aziz et al., 2011a ▶; El Aziz et al., 2011b ▶)
TQ	3mg/kg, i.p., for 5 days	OVA-induced allergy in mice	Reduced IL-4, IL-5, IL-13, IgE, OVA-specific IgE, IgG1, and IgG2a levels Increased IFN-γ	(El Gazzar et al., 2006a ▶)
TQ	3mg/kg, i.p., for 5 days	OVA-induced allergy in mice	Decreased IL-4, IL-5, and IL-13 levels and reduced PGD2 production, COX-2 expression, peribronchial eosinophil infiltration and goblet cells hyperplasia	(El Mezayen et al., 2006 ▶)
TQ	3mg/kg, i.p., for 5 days	OVA-induced allergy in mice	Inhibition of 5-lipoxygenase Reduced total and differential cell count, and diminished levels of LTB4, LTC4, IL-4, IL-5, IL-13, and IL-10 in BALF	(El Gazzar et al., 2006b ▶)

OVA: ovalbumin, BALF: bronchoalveolar lavage fluid, RPMCs: rat peritoneal mast cells preparation, PMN: polymorphonuclear leukocyte, i.p.: intraperitoneal.

**Clinical evidence**

*N. sativa* and its constituents attenuated allergic airway inflammation in animal models of allergic disorders; these effects may be mediated through inhibiting pro-inﬂammatory cytokines, such TNF-α, IL-4, IL-5, IL-13 and IL-1β and down-regulation of PGD_2_ and COX-2 expression as well as reduction of airway inflammatory cell infiltration. Therefore, *N. sativa *supplementation may be a novel strategy for the treatment of various airway inflammatory disorders.

The therapeutic role of *N. sativa* seed on seasonal allergic rhinitis was demonstrated by Ansari et al. In this study, adults with symptomatic seasonal allergic rhinitis were randomized in a single-blind manner to daily receive 250 mg of *N. sativa* seeds orally (3.12 mg/kg/day, for 15 days). All the patients were also assessed for physiological parameters and presence or absence of seasonal allergic rhinitis symptoms. *N. sativa *seeds decreased seasonal allergic rhinitis symptoms score. The difference between patients' systolic and diastolic blood pressure was not statistically signiﬁcant before and after treatment with *N. sativa*, while pulse rate and body temperature significantly decrease from day 0 to day 15 of the study (Ansari et al., 2006 ▶). Işık et al., evaluated the eﬀect of *N. sativa *seed on 31 patients with allergic rhinitis who were only sensitive to house dust mites. Twenty four subjects were considered the experimental group and treated with allergen-specific immunotherapy for one month and 7 patients were included in the placebo group. The experimental group were randomly assigned to receive either *N. sativa* seed supplementation (2 g/day or 25 mg/kg orally) or continuing immunotherapy for 30 days. The diagnosis of allergic rhinitis was done obtaining medical history, physical examination and skin prick test. Allergic rhinitis symptoms were assessed by visual analog scale (VAS) after 30 days of treatment. *N. sativa* improved clinical symptoms and increased phagocytic and intracellular killing activities of polymorphonuclear leukocyte (PMN) and CD8 counts (Işık et al., 2010 ▶). Moreover, the eﬀect of *N. sativa* seed powder and montelukast in patients with seasonal allergic rhinitis were investigated. A total of 47 patients were randomized to receive either *N. sativa* (250 mg/day) or montelukast (10 mg/day) orally for two weeks. *N. sativa* and montelukast significantly reduced daytime, nighttime and ophthalmic symptoms, body temperature, pulse rate and total eosinophil count in both groups. However, montelukast showed drug-related side effects like headache, dizziness and heart burn compared with *N. sativa *(Ansari et al., 2010 ▶). 

A study done by Kalus et al., determined the effect of *N. sativa *oil capsules (40 and 80 mg/kg/day, for 28 days) in patients with allergic disease in two placebo-controlled and two open-label studies. *N. sativa *decreased plasma triglycerides, but increased HDL cholesterol and improved clinical symptoms. However, *N. sativa *did not affect IgE, eosinophil count, lymphocyte subpopulation, endogenous cortisol level and adrenocorticotropic hormone (ACTH) release (Kalus et al., 2003 ▶). In addition, effects of *N. sativa *seed oil against allergic rhinitis were investigated in patients with different severities (mild, moderate and severe). A total of 68 participants aged 6–45 years with allergic rhinitis were included in the study and treated with 2 drops of *N. sativa* oil nasally (one in each nostril) 3 times a day for 6 weeks. The study revealed that 100% of the patients in mild severity group, 68.7% of the moderate severity group, and 58.3% of the severe group had no symptoms after 6-week treatment (Alsamarai et al., 2014 ▶). The therapeutic effects of *N. sativa* oil capsules (0.5 ml/day) in 66 patients with allergic rhinitis, were also evaluated. Patients were randomly assigned to receive either *N. sativa* or placebo. Clinical symptoms were assessed after 4 weeks of treatment. The study revealed that *N. sativa* reduced nasal congestion, sneezing attacks, turbinate hypertrophy and mucosal pallor, rhinorrhea and nasal itching (Nikakhlagh et al., 2011 ▶).

In summary, it was shown that *N. sativa *significantly improved the clinical symptoms of allergic complaints in humans by inhibition of histamine release, metabolites of lipoxygenase pathway like leukotrienes and possibly by non-selective blocking of the histamine receptors, which might explain the beneficial traditional therapeutic use of *N. sativa *in allergic disorders*.* The clinical evidence on anti-allergic effects of *N. sativa* and its constituents are summarized in Table 1b.

**Table 1b:** The clinical evidence on anti-allergic effects of *Nigella sativa* and its constituents

**Plant preparations**	**Dose/route of administration/** **treatment period**	**Study models**	**Effects**	**Reference**
*N. sativa seed* powder	250mg/day, orally, for 15 days	Allergic patients	Improved clinical symptoms No effect on systolic and diastolic blood pressure Decreased pulse rate and body temperature	(Ansari et al., 2006 ▶)
*N. sativa oil*	40 and 80mg/kg/day, orally, for 28 days	Allergic patients	Improved clinical symptoms No significant effect on IgE level and eosinophil count Decreased the plasma triglycerides Increased the HDL cholesterol	(Kalus et al., 2003 ▶)
*N. sativa oil*	15ml/drop, i.n., for 42 days	Allergic rhinitis patients	Improved allergic symptoms	(Mohamed Alsamarai et al., 2014)
*N. sativa seed *powder	2g/day, orally, for 30 days	Allergic rhinitis patients	Improved clinical symptoms Increased phagocytic and intracellular killing activities of PMNs and CD8 counts	(Işık et al., 2010 ▶)
*N. sativa seed powder*	250mg/day, orally, for 14 days	Allergic rhinitis patients	Decreased daytime, ophthalmic, and nighttime symptoms	(Ansari et al., 2010 ▶)
*N. sativa oil*	0.5ml/day, orally, for 28 days	Allergic rhinitis patients	Improved clinical symptoms	(Nikakhlagh et al., 2011 ▶)

i.n.: intranasal.

**The effect of** ***N. sativa*** **and its constituents on asthma**

The anti-asthmatic effects of* N. sativa* and its constituents were evaluated in experimental and clinical studies.

**Experimental evidence**

Preclinical studies using *N. sativa* preparations showed bronchodilatory (Al-Majed et al., 2001 ▶; Boskabady et al., 2008 ▶; El Aziz et al., 2011a ▶; Gilani et al., 2001 ▶; Keyhanmanesh et al., 2013 ▶; Keyhanmanesh et al., 2014a ▶; Saadat et al., 2015 ▶), anti-histaminic (Chakravarty, 1993 ▶; El Aziz et al., 2011a ▶; Saadat et al., 2015 ▶; Saleh et al., 2012 ▶), anti-inflammatory (Abbas et al., 2005 ▶; Balaha et al., 2012 ▶; Büyüköztürk et al., 2005 ▶; Ebrahimi et al., 2016 ▶; El Gazzar et al., 2006b ▶; Fallahi et al., 2016 ▶; Keyhanmanesh et al., 2014c ▶; Keyhanmanesh et al., 2015 ▶; Saleh et al., 2012 ▶; Shahzad et al., 2009 ▶), anti-leukotrienes (Mansour and Tornhamre, 2004 ▶) and immunomodulatory (Abbas et al., 2005 ▶; Balaha et al., 2012 ▶; El Gazzar et al., 2006b ▶; Gholamnezhad et al., 2014 ▶; Gholamnezhad et al., 2015b ▶; Shahzad et al., 2009 ▶) effects in animal models of asthma or in mitogen- stimulated cells. The experimental evidence on *N. sativa* and its constituent's effects on asthma are summarized in Table 2a.

**Clinical evidence**

Nine clinical studies showed potential efficacy of *N. sativa* on asthma outcomes and biomarkers.

The therapeutic efficacy of immunotherapy combined with probiotics and *N. sativa* (15 mg/kg/day) was evaluated in terms of the number of Th17 cells and clinical symptoms of asthma. A total of 31 children with mild asthma were randomized to receive immunotherapy, immunotherapy plus *N. sativa*, immunotherapy plus probiotic, or immunotherapy plus *N. sativa* and probiotic openly for 14 weeks. The result showed that there was no significant difference in the mean number of Th17 cells between pre and post-treatment values among four treatment groups. There was a significant difference in asthma control test (ACT) score before and after treatment with immunotherapy plus *N. sativa*, before and after immunotherapy plus probiotic, as well as before and after immunotherapy plus *N. sativa* and probiotic. A significant relationship between the numbers of Th17 cells and the ACT score was found in all groups. The combination of immunotherapy with *N. sativa *and probiotics did not reduce the number of peripheral blood Th17 cells in mild asthmatic children, but improved the clinical symptoms (Kardani et al., 2013 ▶).

The effect of combination of immunotherapy house dust mite and probiotic or *N. sativa *(15 mg/kg/day) was evaluated with respect to either the induction of CD4^+^CD25^+^foxp3^+^Treg and CD4^+^IL-10^+^ or control of asthma symptoms in mild asthmatic children. Thirty one children with mild asthma were randomized to receive immunotherapy plus placebo, immunotherapy plus probiotic, immunotherapy plus *N. sativa*, or immunotherapy plus probiotic and *N. sativa*, for 14 weeks. The results showed a non-significant decrease in CD4^+^CD25^+^foxp3^+^Treg cell number in all treatment groups, a non-significant increase in CD4^+^IL-10^+^ number in immunotherapy plus placebo group while this marker had a non-significant decrease in the other three groups. All groups except immunotherapy and placebo group, showed a significant increase in ACT score. Adjuvant probiotic or *N. sativa* in immunotherapy, improved asthma symptoms in children with mild asthma (Susanti et al., 2013 ▶).

The prophylactic effect of boiled extract of *N. sativa* (15 ml/kg of 0.1 g %) was examined on asthma. Twenty-nine asthmatic adults were divided into control (14 patients) and study groups (15 patients), and they were followed for 3 months. All asthma symptoms, frequency of asthma symptoms/week, chest wheezing, and pulmonary function tests (PFT) values improved in the study group. The smaller effect of the extract from *N. sativa* on some PFTs, especially on MEF25, may indicate that this plant has little effect on small airways. The results of phase I study generally suggested a prophylactic effect for *N. sativa* on asthma (Boskabady et al., 2007 ▶).

**Table 2a:** The experimental evidence of *N. sativa* and its constituents effects on asthma

	**Plant preparations**	**Dose** **/route of administration/** **treatment period**	**Study models**	**Effects**	**Reference**
*In vitro*	Aqueous-methanol extract of *N. sativa *seeds and its petroleum ether fraction	0.01-10.00 mg/ml	Guinea pig tracheal chains	Inhibition of K^+^-induced contractions in trachea Bronchodilatory and calcium antagonist activities	(Gilani et al., 2001 ▶)
	*N. sativa *fractions	0.8, 1.2, 1.6 and 2.0g%	Guinea pig tracheal chains	Tracheal smooth muscle relaxant effect	(Boskabady et al., 2008 ▶)
	Constituents of 20% *N. sativa *methanolic fraction	50, 100, 150 and 200mg/l	Guinea pig model of asthma	Tracheal smooth muscle relaxant effect	(Keyhanmanesh et al., 2013 ▶)
	TQ	-	Guinea pig tracheal chains	A concentration-dependent decrease in the tension of carbachol-precontracted tracheal smooth muscle	(Al-Majed et al., 2001 ▶)
*In vivo*	*N. sativa*	-	Conalbumin sensitized mice	Reduced blood eosinophil count, IgG1 and IgG2a levels, cytokine profiles and inflammatory cells in lung tissue	(Abbas et al., 2005 ▶)
	*N. sativa *oil	0.5 ml/kg or 2.5 ml/kg (oral, for 3 weeks)	OVA- sensitized guinea pigs	Anti-inflammatory and antioxidant effects	(Saleh et al., 2012 ▶)
	*N. sativa *oil	1 and 4ml/kg/day for 31 day (oral, for 31 days)	OVA-induced mice	Decreased airway hyperresponsiveness, total WBC, macrophages and eosinophils counts, levels of IL-4, IL-5 and IL-13 in BALF, serum levels of total IgE and OVA-specific IgE and IgG1 Increased BALF level of IFN-γ and serum level of OVA-specific IgG2a	(Balaha et al., 2012 ▶)
	*N. sativa *methanolic fraction	3mg/kg (i.p. single dose)	Guinea pig tracheal chains	Decreased tracheal responsiveness to methacholine and OVA, pathological changes and BALF eosinophil	(Keyhanmanesh et al., 2014 ▶)
	TQ	3mg/kg for 5 days (i.p. for 5 days)	OVA- sensitized guinea pigs	Decreased tracheal responsiveness to acetylcholine and histamine	(El Aziz et al., 2011 ▶)
	TQ	3mg/kg/ day for 5 days (i.p. for 5 days)	OVA- sensitized mice	Inhibited 5-lipoxygenase Reduced the levels of LTB4, LTC4 and Th2 cytokines Decreased BALF and lung tissue eosinophilia	(El Gazzar et al., 2006 ▶)
	Alpha-hederin	0.02 mg/kg (i.p. single dose)	OVA- sensitized rats	Decreased the levels of miRNA-126, IL-13 mRNA and pathological changes	(Fallahi et al., 2016 ▶)
	Alpha-hederin	0.02 mg/kg (i.p. single dose)	OVA- sensitized rats	Decreased IL-2 and IL-17 mRNA levels and increased miRNA-133a gene expression	(Ebrahimi et al., 2016 ▶)
	Alpha-hederin	0.3 and 3 mg/kg (i.p. single dose)	OVA- sensitized guinea pigs.	Reduced tracheal responsiveness Decreased total WBC and eosinophil counts	(Saadat et al., 2015 ▶)
	Alpha-hederin	0.3 and 3 mg/kg (i.p. single dose)	OVA- sensitized guinea pigs	Decreased blood levels of IL-4 and IL-17 Increased levels of IFN-γ	(Keyhanmanesh et al., 2015 ▶)

OVA: ovalbumin, BALF: bronchoalveolar lavage fluid, i.p.: intraperitoneal.

The adjuvant effects of *N. sativa* oil (0.1 ml/kg/day, for 14 days) in the management of wheeze associated lower respiratory tract illness (LRTI) in children, were investigated in 84 patients assessed on day 0 and reassessed on days 3, 7, 10 and 14 of treatment by using pulmonary index (PI) and peak expiratory flow rate (PEFR). The therapeutic effect of *N. sativa *oil was seen in the form of decreased PI and increased PEFR (Ahmad et al., 2010 ▶).

In a randomized, double-blind, placebo-controlled trial, the effect of *N. sativa* oil supplementation was investigated in terms of clinical and inflammatory parameters of asthma. *N. sativa* oil (500 mg capsules given twice daily for 4 weeks) showed a significant improvement in mean ACT score, a significant reduction in blood eosinophils, and a non-significant improvement in the value of forced expiratory volume in first second (FEV1). Total IgE level did not show any significant changes. This study demonstrated that *N. sativa* oil supplementation improves asthma control as shown by improved pulmonary function with an acceptable safety and tolerability profile among adult asthmatic patients (Koshak et al., 2017 ▶).

In a single-blind, placebo-controlled, randomized study, the effect of *N. sativa* supplementation (1 and 2 g/day for 3 months) with inhaled maintenance therapy was evaluated on inflammation of the airways and limitation of airflow in partly controlled asthma patients. *N. sativa* 2 g/day significantly increased forced expiratory flow (FEF)25-75% and FEV1 (% predicted) after 6 and 12 weeks of treatment. PEFR variability was significantly improved with both doses of *N. sativa* after 6 and 12 weeks of treatment as compared to the controls. Both doses of *N. sativa* produced a significant decrease in fractional exhaled nitric oxide (FeNO) and serum IgE after 12 weeks. A significant increase in the serum IFN-γ at week 12, and a significant improvement in the ACT score at weeks 6 and 12 vs baseline, were also reported (Salem et al., 2017 ▶).

The efficacy of *N. sativa* seed (2 g/day for 3 months) and bee’s honey (a teaspoonful daily for 3 months) were investigated for treatment of asthma. Pulmonary function showed a significant increase in FVC in asthmatics and a significant increase in PEFR in non-asthmatics, while the FEV1 remained unchanged in both groups. This study showed that *N. sativa* in combination with bee’s honey improves pulmonary function in asthmatics with no hepatorenal toxicity (Ameen et al., 2011 ▶).

In a comparative study, the bronchodilatory effects of 21-day administration of* N. sativa* and *Anthemis- nobilis* (chamomile) were evaluated in 54 patients with chronic bronchial asthma. Inhalation of boiled extract of the plants (100 mg/kg) for 5-10 min (using a vapor machine) showed a significant elevation in the values of FEV1 (% predicted) and forced volume capacity (FVC), and a significant reduction in asthmatic attacks. Symptomatic improvement following *N. sativa* administration was more marked than that caused by chamomile (Al-Jawad et al., 2012 ▶).

The bronchodilatory effects of the oral administration of boiled extract of *N. sativa* (50 and 100 mg/kg, for 4 days) in comparison with oral theophylline (syrup, 6 mg/kg), were studied in 15 asthmatic patients. The results showed that the extract caused significant increases in all measured PFTs, in most time intervals. However, the increase in forced expiratory volume in first second (FEV1), maximal mid-expiratory flow (MMEF) and MEF50 following administration of both doses of boiled extract and increase in MEF75 and MEF25 following administration of its lower dose were significantly lower than those of theophylline. The onset of bronchodilatory effect of extract was similar to that of theophylline (i.e. 30 min), and the effect of extract declined after 150 min following administration which was comparable to that of theophylline. The effect of both doses of the extract was also significantly less than that of inhaled salbutamol (200 mg) 30 min post-administration. The results of this study showed that *N. sativa* has a relatively potent bronchodilatory effect on asthmatic airways. However, the effect of boiled extract of this plant on most of the measured PFTs parameters was less than those of theophylline (at the used concentrations) (Boskabady et al., 2010 ▶).

Preclinical studies showed bronchodilatory, smooth muscle relaxant, spasmolytic, anti-histaminic, anti-inflammatory, anti-leukotrienes, and immunomodulatory effects for *N. sativa* fractions, thymoquinone and alpha-hederin in animal models of allergic asthma. In clinical studies, *N. sativa* increased FEF25-75%, FEV1%, FVC and IFN-γ, but decreased FeNO, IgE and eosinophils counts in the blood and improved clinical symptoms and PFTs in asthmatic patients. In addition, the plant reduced the required dose of inhaler and oral β-agonists, inhaler and oral corticosteroid, and oral theophylline. The extract of *N. sativa* also showed bronchodilatory effect similar to that of theophylline in asthmatic patients. The clinical effects of *N. sativa* and its constituents on asthma are summarized in Table 2b.

**Table 2b:** The clinical evidence of *N. sativa* and its constituents effects on asthma

**Plant preparations**	**Dose/route of administration/** ** treatment period**	**Study models**	**Effects**	**References**
*N. sativa* powder and IM	15mg/kg/day (oral)	Children with mild asthma	Improved clinical symptoms No effect on the Th17 cell number	(Kardani et al., 2013 ▶)
*N. sativa* powder and IM	15mg/kg/day (oral)	Children with mild Asthma	Improved clinical symptoms No effect on CD4^+^ CD25^+^ foxp3^+^Treg and CD4^+^ IL-10^+^	(Susanti et al., 2013 ▶)
Boiled aqueous extract	15mg/kg/day of 0.1g% (oral)	Asthmatic patients	Improved all asthmatic symptoms, asthma symptom/week, chest wheeze, and PFT values Reduced the required dose of inhaler and oral β-agonists, inhaler and oral corticosteroid, and oral theophylline	(Boskabady et al., 2007 ▶)
*N. sativa* oil	0.09mg/kg/day (oral)	Asthmatic patients	Decreased pulmonary index Improved PEFR	(Ahmad et al., 2010 ▶)
*N. sativa *oil	1000mg/day (13mg/kg/day) (oral)	Asthmatic patients	Reduced eosinophils in blood Improved PFT No effect on the total serum IgE level	(Koshak et al., 2017 ▶)
*N. sativa seed *powder	1 and 2g/day (13 and 26mg/kg/day) (oral)	Asthmatic patients	Improved PFT and ACT score Increased FEF25-75% and FEV1% Decreased FeNO and IgE and increased IFN-γ	(Salem et al., 2017 ▶)
*N. sativa seed *powder	2g/day (26mg/kg/day) (oral)	Asthmatic patients	Increased FVC No effect on FEV_1_	(Ameen et al., 2011 ▶)
Boiled aqueous extract	100 mg/kg (inhalation)	Asthmatic patients	Improved clinical symptoms Elevated FEV1% and FVC/L	(Al-Jawad et al., 2012 ▶)
Boiled aqueous extract	50 and 100 mg/kg/day (oral)	Asthmatic patients	Lower effectiveness on FEV_1_, PEFR, MMEF, MEF_75_, MEF_50_, MEF_25_, and sGaw than theophylline	(Boskabady et al., 2010 ▶)

PFT: pulmonary function test, FEV_1_: forced expiratory volume in first second, FVC: forced volume capacity, PEFR: peak expiratory ﬂow rate, MMEF: maximal mid-expiratory ﬂow, MEF: maximal expiratory ﬂow, sGaw: speciﬁc airway conductance, IM: immunotherapy, Th: T helper, foxp3: factor forkhead box P3, Treg: Regulatory T, PMN: polymorphonuclear leukocyte, ACT: Asthma control test, FeNO: fractional exhaled nitric oxide.

**The effect of** ***N. sativa*** **and its constituents on other respiratory diseases**

Several studies reported the effects of *N. sativa* and its constituents on different respiratory diseases in cellular and animal models as well as clinical studies.

**Experimental evidence**

Some *in vitro* and *in vivo* experiments used human cell lines related to respiratory diseases, for evaluating the effects of *N. sativa* extract and oil as well as its main constituent, TQ. Treatment of human epithelial type 2 (HEp-2) cell with a single dose of TQ (5 µM) led to reduction of cell numbers after 24 hr (Womack et al., 2006 ▶). Anticancer effects of two constituents of *N. sativa*, alpha-hederin and TQ, on HEp-2 cells were also examined. Cytotoxic effect TQ was more than alpha-hederin against HEp-2 cells (Rooney and Ryan, 2005 ▶).

The TQ activity on cell lines related to non-small cell lung cancer (NSCLC) and small cell lung cancer (SCLC) was also examined. The results showed induction of apoptosis, inhibition of cell proliferation and reduction of cell viability at following treatment with TQ 100 μM (Jafri et al., 2010 ▶). Similarity, anti-tumor and anti-metastatic activities of TQ against NSCLC were reported (Attoub et al., 2013 ▶; Ulasli et al., 2013 ▶; Yang et al., 2015 ▶). In addition, subcutaneous (s.c.) injection of NSCLC cell line to mice created a xenograft model to evaluate *in vivo* activity of TQ. Significant reduction of tumor volume and weight was observed after treatment of animal with 5 and 20 mg/kg TQ (s.c.) (Jafri et al., 2010 ▶). 

The alcoholic extract and oil of *N. sativa* were investigated in a human lung cancer cells model. In this model, treating the lung cancer cells with *N. sativa* extract and oil (0.01 to 1 mg/ml) significantly reduced viability of the cells (Al-Sheddi et al., 2014 ▶). In addition, inhibitory effect of the essential oil of *N. sativa* (5.8 mg/ml) on human neutrophil elastase (HNE) activity was shown suggesting the therapeutic effect of the plant on lung damages caused by some diseases including chronic obstructive pulmonary disease (COPD) and emphysema (Kacem and Meraihi, 2006 ▶). 

Other studies used animal models of respiratory disorders to examine the effect of* N. sativa* extract and its active compounds specially TQ. The preventive effect of hydro-ethanolic extract of *N. sativa* (0.08 g/kg/day, in drinking water, for 14 days) on tracheal responsiveness and lung inflammation was shown in a guinea pig model of lung injury induced by sulfur mustard (Boskabady et al., 2011 ▶; Hossein et al., 2008 ▶). The effects of *N. sativa* oil (1ml/kg/day, administered by gavage) and TQ (5 mg/kg/day, i.p., for 5 weeks) were examined against lung fibrosis induced by bleomycin, in rats. Similarly, therapeutic effect of oral administration of TQ (20 and 40 mg/kg/day) for 28 days in a mouse model related to pulmonary fibrosis induced by paraquat was evaluated. The results of these investigations indicated that *N. sativa* oil and TQ produce preventive and therapeutic effects on pulmonary fibrosis through inhibition of oxidative stress and nuclear factor (NF)-kB as well as down-regulation of profibrotic genes (Abidi et al., 2017 ▶; El-Khouly et al., 2012 ▶; Pourgholamhossein et al., 2016 ▶). Aspiration lung injury in a rat model was also improved after treatment with *N. sativa* oil (400 mg/kg/day, administered by gavage for 7 days) by inhibition of lung inflammatory responses, interstitial fibrosis and alveolar edema (Kanter, 2009 ▶). Similarity, the protective effect of TQ against lung injury induced by chronic exposure to toluene was shown in rats. In this model, TQ (50 mg/kg/day, administered orally for 12 weeks) inhibited the pulmonary inflammatory responses, alveolar edema, interstitial fibrosis and necrosis formation (Kanter, 2011 ▶).

In a guinea pig model of COPD, pretreatment of cigarette smoke-exposed animal with hydroethanolic extract of *N. sativa* (0.1 g/kg/day, in drinking water for 3 months) led to a preventive effect on tracheal responsiveness to methacholine and ovalbumin (Keyhanmanesh et al., 2014b ▶).

Gunes et al. (2017) ▶ investigated the effect of TQ treatment (50 mg/kg/day, administered by gavage for 5 days) on lung tissue injury induced by hyperbaric oxygen (HBO₂) therapy, in a rat model. The antioxidant property of TQ led to reduction of lipid hydroperoxide (LOOH) and total sulfhydryl group (-SH) causing a preventive effect on HBO₂-induced lung injury (Gunes et al., 2017 ▶). In another study, the effects of *N. sativa *oil (4 ml/kg/day, i.p.) on hyperoxia-induced lung injury were assessed in rats. Reduction of oxidant biomarkers, increment of antioxidant agents and reduction of lung damage severity after treatment with *N. sativa oil* were observed (Tayman et al., 2013 ▶).

In a rabbit model with bacterial rhinosinusitis, *N. sativa* extract (50, 100, 200 mg/kg/day, administered orally for 7 days) reduced nitric oxide (NO) level and thus, prevented hisopathological changes (Yoruk et al., 2017 ▶). Activity of *N. sativa* ethanolic extract (125, 250, 500 mg/kg/day, administered by gavage) against lung injury caused by cecal ligation and puncture (CLP) in a sepsis rat model (sepsis is a serious infection) led to reduction of pro-inflammatory cytokines, oxidative stress markers and histopathological insults. Therefore, this plant could be of therapeutic value for inhibiting of formation of CLP-induced sepsis (Bayir et al., 2012 ▶). 

Oxidative stress caused by cyclophosphamide (CP), an anticancer drug, caused lung injury. In a study, TQ (100mg/kg/day, administered orally for 14 days) was used to improve CP-induced pulmonary damage in a rat model. The antioxidant and anti-inflammatory properties of TQ led to protective effect of this component in healthy lung cells (Suddek et al., 2013 ▶). TQ (8, 12, and 16 mg/kg/day, i.p. for 2 weeks) also reduced pulmonary artery hypertension induced by monocrotaline through blocking pulmonary arterial remodeling in a rat model (Zhu et al., 2016 ▶). The preventive effect of TQ (6 mg/kg, i.p. administered twice before exposure to diesel exhaust particles (DEP)) on airway resistance was shown in a mouse model of acute exposure to DEP (Nemmar et al., 2011 ▶). In another study, antispasmodic effect and increase in mucociliary clearance were induced by nigellone but not with TQ in a rat model (Wienkotter et al., 2008 ▶). The effects of *N. sativa* and its constituents in different cell lines and animal models related to respiratory diseases, are summarized in Table 3a.

**Clinical evidence**

There are some clinical studies on the effect of *N. sativa* and its constituents in patients with respiratory disorders. A randomized double-blinded placebo-controlled trial showed a protective effect for aqueous extract of *N. sativa* in chemical war victims after administration of a single dose (187 mg/kg/day of 50 g%) of extract for 2 months. In this trial, all respiratory parameters such as pulmonary functional test (PFT) values, respiratory symptoms and chest wheeze significantly improved in treated patients. In addition, use of drugs such as oral β-agonists and corticosteroid in victims was reduced after administration of extract of *N. sativa* compared to untreated patients (Boskabady et al., 2008 ▶). 

In a crossover randomized controlled trial, improvement of nasal dryness, obstruction and crusting related to age was observed after treatment of geriatric patients with *N. sativa* oil (one puff per nostril included 22.6 µg of *N. sativa* oil in 25µl per nostril) compared to isotonic sodium chloride solution (ISCS). Of both *N. sativa* oil and ISCS, three puffs were administered in each nostril 3 times daily. The results of this trial suggested that *N. sativa* oil could be a more effective drug than ISCS for symptomatic relief from aging-induced symptoms (Oysu et al., 2014 ▶).

In another trial done by Dirjomuljono et al. (2008) ▶, in 186 acute tonsillo-pharyngitis patients, the clinical effectiveness of a combination of *N. sativa* and *Phyllanthus niruri* extract (NSPN extract) was evaluated. In this randomized, double-blind, placebo-controlled study, NSPN capsules (containing 360 mg *N. sativa* and 50mg *P. niruri* extracts) were orally administered 3 times daily for 7 days. At the end of study, the sore throat was completely relieved in patients treated with NSPN extract compared to placebo group (Dirjomuljono et al., 2008 ▶).

**Table 3a:** The experimental evidence of *Nigella sativa* and its constituents effects on various respiratory diseases

	**Plant preparations**	**Dose** **/route of administration/treatment period**	**Study models**	**Effects**	**Reference**
***In vitro*** ** studies**	Alcoholic extract and oil	0.01 to 1mg/ml	Human lung cancer cellular model	Reduced cell viability	(Al-Sheddi et al., 2014 ▶)
	TQ	5microM	HEp-2 cellular model	Reduced cell numbers	(Womack et al., 2006 ▶)
	Alpha-hederin TQ	6-40µM 25-150µM	HEp-2 cellular model	Inhibited cell proliferation Elicited apoptosis and necrosis	(Rooney and Ryan, 2005 ▶)
	TQ	100μM	NSCLC and SCLC models	Induced apoptosis Inhibited cell proliferation Decreased cell viability	(Jafri et al., 2010 ▶)
	TQ	1–100μM	NSCLC	Inhibited cell viability Anticancer activity	(Attoub et al., 2013 ▶)
	TQ	5μM	NSCLC	Induced apoptosis via down-regulated NF-kB and Bcl-2 Anticancer activity	(Ulasli et al., 2013 ▶)
	TQ	0, 5, 10, 20, 40, 80, 160μmol/L	NSCLC	Inhibited cell proliferation Anti-metastatic activity	(Yang et al., 2015 ▶)
***In vivo *** **studies**	TQ	5 and 20mg/kg (injected s.c.)	Xenograft model using NSCLC	Reduced volume and weight of tumor	(Jafri et al., 2010 ▶)
	Hydro-ethanolic extract	0.08g/kg/day (in drinking water for 14 days)	Sulfur mustard- exposed guinea pig model	Preventive effect TR and lung inflammation	(Boskabady et al., 2011 ▶; Hossein et al., 2008 ▶)
	Hydro-ethanolic extract	0.1g/kg/day (in drinking water for 3 month)	Cigarette smoke-exposed guinea pig	Preventive effect on TR	(Keyhanmanesh et al., 2014 ▶)
	Hydro-ethanolic extract	50, 100, 200mg/kg/day (administered orally for 7 days)	Bacterial infection induced rhinosinusitis	Reduced NO level Prevented histopathological changes	(Yoruk et al., 2017 ▶)
	Ethanolic extract	125, 250, 500mg/kg/day (administered by gavage)	CLP induced sepsis	Reduced pro-inflammatory cytokines Reduced oxidative stress markers Reduced histopathological changes	(Bayir et al., 2012 ▶)
	*N. sativa *oil	1ml/kg/day (administered by gavage)	Bleomycin induced pulmonary fibrosis	Reduced inflammatory index and fibrosis score Prevented pulmonary fibrosis	(Abidi et al., 2017 ▶)
	TQ	20 and 40mg/kg/day (administered orally for 28 days)	Paraquat induced pulmonary fibrosis	Inhibited oxidative stress Down-regulated pro-fibrotic genes Prevented pulmonary fibrosis	(Pourgholamhossein et al., 2016 ▶)
	TQ	5 mg/kg/day (injected i.p. for 5 weeks)	Bleomycin induced pulmonary fibrosis	Inhibited NF-kB Prevented pulmonary fibrosis	(El-Khouly et al., 2012 ▶)
	*N. sativa *oil	400 mg/kg/day (administered by gavage for 7 days)	Aspiration lung injury	Inhibited lung inflammatory responses, Inhibited interstitial fibrosis Inhibited alveolar edema	(Kanter, 2009 ▶)
	TQ	50mg/kg/day (administered orally for 12 weeks)	Toluene-exposed rat model	Inhibited pulmonary inflammatory Inhibited alveolar edema Inhibited interstitial fibrosis Inhibited necrosis formation	(Kanter, 2011 ▶)
	TQ	50mg/kg/day (administered by gavage for 5 days)	HBO₂-induced lung injury	Reduced LOOH and SH levels Prevented lung injury	(Gunes et al., 2017 ▶)
	*N. sativa* oil	4ml/kg/day (injected i.p.)	Hyperoxia-induced lung injury	Reduced oxidant biomarkers Increased antioxidant agents Reduced severity of lung damage	(Tayman et al., 2013 ▶)
	TQ	100mg/kg/day (administered orally for 14 days)	CP-induced pulmonary injury	Attenuated pro-inflammatory cytokine and TNF-α Alleviated histopathological changes	(Suddek et al., 2013 ▶)
	TQ	8, 12, 16mg/kg/day (injected i.p. for 2 weeks)	Monocrotaline-induced pulmonary artery hypertension	Inhibited pulmonary arterial remodeling Improved pulmonary artery hypertension	(Zhu et al., 2016 ▶)
	TQ	6mg/kg (injected i.p. twice before exposure with DEP)	DEP- exposed mouse model	Prevented airway resistance	(Nemmar et al., 2011 ▶)

TQ: thymoquinone; HEp-2: human epithelial type 2; NSCLC: non-small cell lung cancer; SCLC: small cell lung cancer; CLP: cecal ligation and puncture; HBO₂: hyperbaric oxygen; CP: cyclophosphamide; DEP: diesel exhaust particles; TR: tracheal responsiveness; LOOH: lipid hydroperoxide; SH: sulfhydryl group; PFT: pulmonary function test; NO: nitric oxide; NF-kB: nuclear factor, s.c.: subcutaneous; i.p.: intraperitoneal.

Reviewed studies showed a relatively potent preventive and therapeutic effect for various preparations derived from* N. sativa* on various respiratory diseases. Preclinical studies using animal or cellular models of respiratory diseases showed anticancer, anti-metastatic, anti-inflammatory, antioxidant, anti-infection and anti-fibrotic effects for extract and oil of *N. sativa* as well as TQ. Clinical studies also indicated improvement of respiratory symptoms and PFT in chemical war victims as well as controlled clinical symptoms in tonsillo-pharyngitis patients treated by *N. sativa*. In addition, symptomatic relief from nasal dryness, obstruction and crusting in geriatric patients were observed following treatment with this plant. The clinical effects of *N. sativa* and its constituents on various respiratory diseases are summarized in Table 3b. 

**Table 3b:** The clinical evidence of *Nigella sativa* and its constituents effects on various respiratory diseases

	**Plant preparations**	**Dose** **/route of administration/treatment period**	**Study models**	**Effects**	**Reference**
**Clinical evidence**	Boiled aqueous extract	18.7mg/kg/day of 50g%) (administered orally for 2 months)	Chemical war victims	Decreased the use of inhaler and oral β-agonists and oral corticosteroid in the study group Improved PFT values, respiratory symptoms and chest wheeze	(Boskabady and Farhadi, 2008 ▶)
	*N. sativa *oil	1.808µg/kg/day (administered nasally for 2 weeks)	Patients with nasal dryness	Improved nasal dryness, obstruction and crusting No effect on mucociliary clearance, nasal burning and itching	(Oysu et al., 2014 ▶)
	*N. sativa *seed powder	14.4mg/kg/day (administered orally for 7 days)	Tonsillo-pharyngitis patients	Improved clinical symptoms	(Dirjomuljono et al., 2008 ▶)

## Discussion

All reviewed studies demonstrated that supplementation or treating patients with* N. sativa* seed (25-250 mg/kg/day) or oil (25 µl-0.5 ml/day) for 15-30 days, alleviates symptoms of allergic rhinitis and decreases the body temperature in allergic patients. These effects may be related to different immunomodulatory properties of the plant including enhancing the phagocytic and intracellular killing activities of PMN and increment of CD8 counts as well as antihistaminic activities of *N. sativa* lipid- and water-soluble constituents.

In adult patients with asthma, supplementary or single administration of *N. sativa* seed (1-2 g/day), boiled extract (50-100 mg/kg), or oil (1g/day) for 3-12 weeks significantly improved asthma symptoms and pulmonary function test, while in some experiments, non-significant effects on some variables including MEF25, and FEV1 was observed. *N. sativa *decreased blood eosinophils, FeNO and IgE levels and serum IFN-γ in one study; however, in another experiment, IgE level was not changed significantly. In mild to moderate asthmatic children combination of immunotherapy house dust mite with* N. sativa* seed (15 mg/kg/day, 14 weeks) or oil (0.1 ml/kg/day, 2 weeks) improved asthma control test and asthma symptoms, while immunological changes such as decrease CD4^+^CD25^+^foxp3^+^Treg, increase CD4+IL-10+, and Th17 cells were not affected.

Antioxidant, anti-inflammatory and antitumor effects of *N. sativa* seed, extracts, oil and its main constituent TQ were established in different animal experimental models and human cancer cell lines. *N. sativa* oil (0.01 to 1 mg/ml) and TQ (5-100 μM) showed antitumor effects and reduced HEp-2 cells, NSCLC, and SCLC and lung cancer cell numbers. In different animal models of lung injury and inflammation, aspiration lung injury, toxins (sulfur mustard, paraquat, bleomycin, toluene, cigarette, CP, DEP) induced lung injury, HBO₂ therapy, bacterial rhinosinusitis, sepsis, pulmonary artery hypertension, the protective effects of *N. sativa *and TQ were demonstrated. These studies showed that treating animal with* N. sativa *or TQ inhibited lung inflammatory responses, oxidative stress and NF-kB, reduced interstitial fibrosis, alveolar edema, profibrotic genes expression and inflammatory cytokines. In addition, therapeutic effects of *N. sativa *extract (187 mg/kg/day of 50 g%) in chemical war victims in terms of improvement of PFT values, respiratory symptoms and chest wheeze, were shown. Nostril spray of *N. sativa *oil (22.6 µg or 25 µl) in geriatric patients resulted in relief from nasal dryness, obstruction and crusting. NSPN extract (360 mg *N. sativa* and 50 mg *P. niruri* extracts) in patients with acute tonsillo-pharyngitis also relieved sore throat.

In conclusion, considering the above-mentioned bronchodilatory, anti-inflammatory, antioxidant and immunomodulatory effects,* N. sativa* and its constituents may be regarded as an effective remedy in allergic and obstructive lung diseases as well as other respiratory diseases as a preventive and/or relieving therapy. However, more standard clinical trials and human studies should be designed in future in order to reach a better scientific judgment before production of *N. sativa* based drugs. 
